# Laboratory Data and IBDQ—Effective Predictors for the Non-Invasive Machine-Learning-Based Prediction of Endoscopic Activity in Ulcerative Colitis

**DOI:** 10.3390/jcm12113609

**Published:** 2023-05-23

**Authors:** Otilia Gavrilescu, Iolanda Valentina Popa, Mihaela Dranga, Ruxandra Mihai, Cristina Cijevschi Prelipcean, Cătălina Mihai

**Affiliations:** 1 Medicale I Department, “Grigore T. Popa” University of Medicine and Pharmacy, 700115 Iasi, Romania; otilianedelciuc@yahoo.com (O.G.);; 2 “Saint Spiridon” County Hospital, 700111 Iasi, Romania; cristinacijevschi@yahoo.com; 3 Medicale II Department, “Grigore T. Popa” University of Medicine and Pharmacy, 700115 Iasi, Romania; ruxandra.mihai11@gmail.com

**Keywords:** ulcerative colitis, disease activity, non-invasive biomarkers, quality of life, machine learning

## Abstract

A suitable, non-invasive biomarker for assessing endoscopic disease activity (EDA) in ulcerative colitis (UC) has yet to be identified. Our study aimed to develop a cost-effective and non-invasive machine learning (ML) method that utilizes the cost-free Inflammatory Bowel Disease Questionnaire (IBDQ) score and low-cost biological predictors to estimate EDA. Four random forest (RF) and four multilayer perceptron (MLP) classifiers were proposed. The results show that the inclusion of IBDQ in the list of predictors that were fed to the models improved accuracy and the AUC for both the RF and the MLP algorithms. Moreover, the RF technique performed noticeably better than the MLP method on unseen data (the independent patient cohort). This is the first study to propose the use of IBDQ as a predictor in an ML model to estimate UC EDA. The deployment of this ML model can furnish doctors and patients with valuable insights into EDA, a highly beneficial resource for individuals with UC who need long-term treatment.

## 1. Introduction

Ulcerative colitis (UC) is a chronic inflammatory condition characterized by a recurring pattern of disease flare-ups followed by periods of remission.

When intestinal inflammation is not appropriately managed, UC has an increased risk of complications. Modern treatment strategies for UC have redefined the therapeutic goal as obtaining “disease clearance” with clinical and biological remission as well as endoscopical and histological healing. In the future, molecular remission may also be targeted [1].

Endoscopy represents the gold standard of diagnosis for UC patients. It is a feasible procedure that has the ability to obtain mucosal biopsies and offers high diagnosis accuracy. Endoscopy plays an integral role in the diagnosis, monitoring and management of UC patients [2]. It can assess and stratify disease activity, evolution and treatment [3]. However, endoscopy is invasive and very discomforting for the patient, which makes it challenging for patients to comply with medical recommendations regarding disease monitoring and screening [4]. For this reason, the international community of GI experts is still searching for the ideal biomarker (non-invasive, non-expensive, readily available) to revolutionize disease monitoring in UC [5].

Over the past few years, machine learning (ML) has become a potent instrument in the field of medicine, thanks to its capacity for discrimination and decision making. ML algorithms possess the ability to continually update their learning and capture connections between variables, making them an effective strategy for developing prediction models for UC disease activity. Earlier investigations have revealed that ML models can achieve higher levels of accuracy in evaluating and estimating disease activity [6].

Most of the previous studies that evaluated ML solutions for estimating disease activity have primarily relied on symptoms, laboratory values or radiological and imaging features as predictors [7,8,9].

To the best of our knowledge, there have not been any prior investigations that have utilized ML models to forecast endoscopic disease activity (EDA) in patients with UC incorporating a quality of life assessment tool—the Inflammatory Bowel Disease Questionnaire (IBDQ) score [10]—as one of the predictors. Patient-reported outcomes (PROs) measure the patient’s experience of the disease and impact of treatment without the burden of extensive medical evaluations, leading to improvement in the patient’s quality of life. In recent times, the attainment of living remission, which includes normalized health-related quality of life and the elimination of disability, has emerged as a therapeutic target and disease activity index [11,12].

Our study aimed to (1) develop an easily accessible, economical and non-invasive ML method to accurately predict EDA based on low-cost biological predictors and (2) prove that adding a no-cost clinical predictor such as the IBDQ score can significantly improve the accuracy of estimating endoscopic activity. The deployment of this machine learning forecasting model can furnish doctors and patients with valuable insights into EDA, a highly beneficial resource for individuals with ulcerative colitis who need long-term treatment.

## 2. Materials and Methods

### 2.1. Study Design

We conducted a prospective, observational, single-center, cohort study that included 187 UC patient records. All patients were hospitalized between October 2012 and November 2022 and were confirmed to have a diagnosis of UC using complete hematological, biochemical tests, endoscopic and imagistic examinations and histopathology confirmation. The inclusion criteria required that patients undergo colonoscopy to assess the disease. Each patient completed the IBDQ, which evaluates the health-related quality of life of inflammatory bowel disease (IBD) patients.

Cases were excluded if they had concurrent disorders, such as infections, autoimmune and inflammatory conditions, cirrhosis, neoplasia or hemodialysis, which could influence medical parameters.

The study was approved by the local ethics committee within “Grigore T. Popa” University of Medicine and Pharmacy, Iasi, Romania (No. 65/04.10.2012). Each patient provided written informed consent.

### 2.2. Data Acquisition

The parameters collected for the study comprised multiple continuous variables (IBDQ score, age, smoking pack years, relapses per year, hemoglobin, hematocrit, mean corpuscular volume—MCV, mean corpuscular hemoglobin concentration—MCHC, platelet count, white blood cell count—WBC, erythrocyte sedimentation rate—ESR, fibrinogen, C-reactive protein—CRP, total proteins, albumin) and several binary variables (denoting whether the patient was administered 5-aminosalicylic acid—5-ASA, topical or oral therapy, intravenous or oral corticosteroids, Azathioprine, Infliximab, Adalimumab, or antibiotics).

GI physicians from the Gastroenterology and Hepatology Institute, Iași, Romania, conducted colonoscopies using the EVIS EXERA II endoscopy system (Olympus America, Bartlett, TN, USA). The endoscopic Mayo score was calculated according to the latest European consensus guidelines [13]. We used two classifications for the predictions. For the first classification, a Mayo score of 0 or 1 is considered to indicate endoscopic remission, while a score of 2 or 3 indicates active disease (or relapse). Moreover, a Mayo score of 3 defines severe disease. A Mayo score of 2 indicates moderate endoscopic disease. Finally, we define mild disease (including disease in remission) as an endoscopic Mayo score of 1 or 0.

### 2.3. Preprocessing

The preprocessing, feature selection and models’ implementation were carried out in RStudio 2023.03.0 + 386 (3c53477a, 9 March 2023) for Windows.

Documented continuous variables were normalized in the range [0, 1]. Values of hemoglobin and hematocrit were determined to resolve the differences between sexes.

### 2.4. Feature Selection

As the continuous parameters did not have a normal distribution, the Kruskal–Wallis rank sum test was conducted to identify which of the collected continuous variables were correlated with the endoscopic activity.

Pearson’s chi-squared test of independence was performed to examine whether there was a relationship between each categorical parameter and the endoscopic activity.

The continuous and categorical parameters presenting a significant statistical difference (*p* < 0.5) between the endoscopic classes (activity/remission), as identified using the Kruskal–Wallis and Pearson’s chi-squared tests, were selected as predictors for the ML models.

### 2.5. Development of the ML Models

We randomly split the initial data of 187 UC patients into a training set of 150 records (80%) and a test set of 37 records (20%).

Random forest (RF) and multilayer perceptron (MLP) classifiers were developed. We used the caret::train function in RStudio to build the models. To avoid overfitting, 10-fold cross-validation was employed. To address the issue of imbalanced Mayo classes, we utilized the synthetic minority over-sampling technique (SMOTE) in conjunction with the caret::train function. For the sake of the study’s reproducibility, Table 1 presents the parameters passed on to the caret::train function.

The first RF and the first MLP binary models were developed to predict EDA (activity/remission) based on the selected variables, excluding IBDQ score. The second RF and the second MLP binary classifiers were built to estimate EDA based on all the selected predictors, including the IBDQ score.

The third RF and the third MLP multiclass models were developed to predict endoscopic disease severity (mild/moderate/severe) based on the selected variables, excluding IBDQ score. The fourth RF and the fourth MLP multiclass classifiers were built to estimate endoscopic disease severity based on all the selected predictors, including IBDQ score.

The classifiers that included the IBDQ score as a predictor were built in order to assess whether including easily accessible and cost-free clinical data such as the IBDQ score next to the biological predictive variables could improve endoscopic activity estimation.

We evaluated the ML models for classification accuracy on both the test and training sets. Where applicable, we determined the area under the receiver operating characteristic curve (AUC) (binary AUC for the first and second RF and MLP models and multiclass AUC for the third and fourth RF and MLP models), sensitivity, specificity and the positive (PPV) and negative predictive value (NPV).

## 3. Results

The study included 187 UC patients, of which 110 (59%) were male and 77 (41%) were female. The age of the participants ranged from 18 to 76 years. Table 2 summarizes the selected parameters and IBDQ score for all patients as well as for each endoscopic activity class. Continuous variables are represented as median (interquartile range) and categorical variables are illustrated by the number of occurrences of each category. Endoscopic activity and severity classes were imbalanced in size (as illustrated in Table 2), which prompted the use of SMOTE in the development of the models.

Kruskal–Wallis rank sum (Table 3) and chi-square (Table 4) tests were applied to identify the continuous and categorical variables that were significantly related to the endoscopic Mayo score.

The feature selection step identified the following predictors to be used for the training of the ML models (*p* < 0.05): platelet count, WBC, fibrinogen, CRP, administration of oral corticosteroids and IBDQ score.

Based on the variables selected as predictors by the feature selection step, eight ML models were trained.

We developed the first RF and MLP models to predict EDA based on selected variables, excluding the IBDQ score. Table 5 presents a comparative overview of the performance metrics achieved by these classifiers.

The second RF and the second MLP classifiers were built to estimate EDA based on all the selected predictors, including the IBDQ score. The performance metrics achieved by these classifiers are comparatively presented in Table 6.

**Figure 1:**
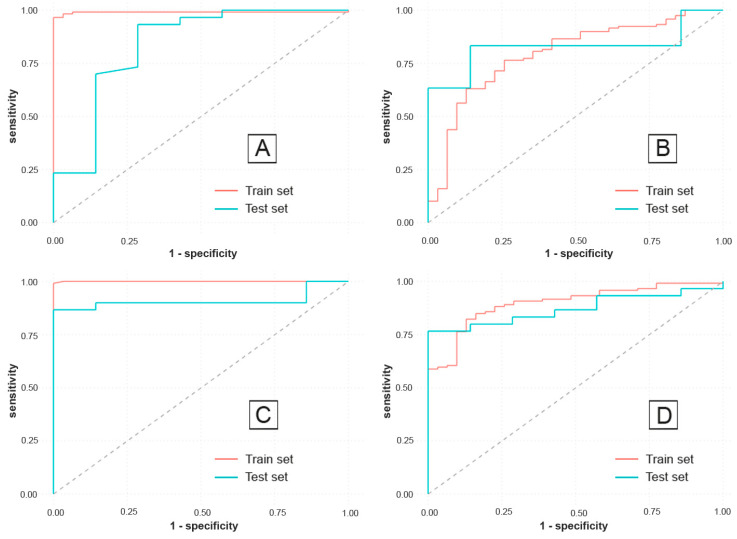
(**A**) ROC curve of the first RF model that excluded IBDQ as a predictor. (**B**) ROC curve of the first MLP model that excluded IBDQ as a predictor. (**C**) ROC curve of the second RF model that included IBDQ as a predictor. (**D**) ROC curve of the second MLP model that included IBDQ as a predictor.

Figure 1 shows the ROC curves that illustrate the performance of all four developed models on both the train and test sets. It can be spotted visually how the inclusion of the IBDQ score as a predictor determined an increase in AUC for both the RF and MLP models.

Subsequently, we implemented and tested the third RF and MLP models to predict endoscopic disease severity based on selected variables, excluding the IBDQ score. Table 7 presents a comparative overview of the performance metrics achieved by the third classifiers.

Finally, the fourth RF and the fourth MLP classifiers were built to estimate endoscopic disease severity based on all the selected predictors, including the IBDQ score. The performance metrics achieved by these classifiers are comparatively presented in Table 8.

## 4. Discussion

Our research has produced an ML method that can predict EDA in UC patients using the cost-free IBDQ score and low-cost laboratory data. This is the first study to propose the use of IBDQ scores as a predictor in an ML model aimed to estimate UC EDA. Our findings indicate that it is possible to distinguish between active and inactive UC (with high accuracy) and between mild, moderate and severe disease (with moderate AUC) using non-invasive and low-cost clinical and biological predictors.

Originally, the study went through the feature selection step and identified several non-invasive predictors related to the endoscopic activity: platelet count, WBC, fibrinogen, CRP, administration of oral corticosteroids and IBDQ score. This is in accordance with the existing scientific literature since many of these variables have already been examined in the search for the optimal biomarker for assessing endoscopic activity [14,15,16]. However, our paper is the first to consider IBDQ in conjunction with other biological variables as a trustworthy predictor.

Next, we employed several ML algorithms, RF and MLP, to predict EDA and endoscopic disease severity and comparatively studied their performance. The results show that the inclusion of the IBDQ score in the list of predictors that were fed to the models improved accuracy and AUC for both the RF and the MLP algorithms. Moreover, the RF technique performed visibly better than the MLP method on unseen data (the independent patient cohort). This is good news as, although neural networks are highly powerful predictive tools, RF algorithms are less computationally expensive and are more interpretable, which is necessary for analyzing a large amount of medical data [17].

Distinguishing between active disease and remission in ulcerative colitis is important for monitoring disease progression over time [13,18]. If a patient is in remission, it is important to monitor for signs of relapse [16]. If a patient has active disease, frequent monitoring may be needed to ensure that treatment is effective in inducing remission. The second RF and MLP models achieved high accuracy in distinguishing between active and inactive endoscopic disease. Although useful in clinical practice, the status of active disease or remission does not provide enough detail about the severity of the disease or the potential for complications.

The fourth RF and MLP models were built to push the limit even further by discriminating between mild, moderate and severe disease. This is important because they can help guide treatment decisions and predict disease outcomes [13,16]. For instance, patients with mild disease could be managed through escalation of therapy without a colonoscopy, while more severe disease prompts the need for a colonoscopy. Patients with moderate endoscopic disease may be treated with a combination of medications, while patients with severe endoscopic disease may require more potent medications such as biologics or surgery. The severity of endoscopic disease can also help predict the likelihood of disease complications, such as hospitalization, surgery or colorectal cancer [13]. Patients with severe endoscopic disease are at higher risk for complications and may require closer monitoring. Moreover, clinical trials for ulcerative colitis often stratify patients by endoscopic disease severity, allowing researchers to evaluate the efficacy and safety of different treatments in patients with different levels of disease severity [13]. The accuracy and AUC of the fourth RF and MLP models are, however, mediocre. This is due to the small number of patients in our cohort, which did not allow for exhaustive model training. This limitation will be resolved in our future studies, after enriching our database with more data.

The primary goal of treating patients with IBD, as the Selecting Therapeutic Targets in Inflammatory Bowel Disease initiative (STRIDE-II) states [19], is to maintain long-term health-related quality of life by obtaining clinical response and remission, endoscopic healing, normalization of C-reactive protein, erythrocyte sedimentation rate and calprotectin [19].

The definitive aim of PRO measurement (through questionnaires such as the IBDQ) is to improve the quality of life with treatment so as to be comparable to the quality of life before disease onset. Therefore, a therapeutic target set by STRIDE-II is to achieve favorable PROs, emphasizing the importance of quality of life as a therapeutic goal. The inclusion of a resource-free tool such as the IBDQ as a predictor in ML models to estimate endoscopic outcomes is a natural, scientifically supported step.

Several disease activity biomarkers for UC have been studied and used in clinical practice [18]. Among them, fecal calprotectin (FC) proved to be the best, achieving highest prediction accuracies. FC is a non-invasive and repeatable marker that has been demonstrated to have a good correlation with disease activity, and even to have the ability of indicating mucosal healing in patients with UC [20].

However, this indicator has some disadvantages. Every step of FC measurement can influence the results, leading to misinterpretations. Moreover, there may be false-positive values in patients with inflammatory polyps within 3 months of surgery or within 2 weeks of colonoscopy or in patients taking proton pump inhibitors or non-steroidal anti-inflammatory drugs [18]. Moreover, compared to ML tools for assessing disease activity in patients with UC in routine practice, FC is more expensive and is not routinely available in some countries [18].

ML can be used as a valuable tool for improving patient care at every stage of disease. UC is a progressive disease with an unpredictable course, and it is associated with high healthcare costs. Disease activity is an indispensable assessment for developing and determining appropriate management in patients with UC.

ML algorithms are quickly becoming an integral part of modern healthcare, being affordable, non-invasive and easy to use. ML algorithms could help reduce the substantial costs associated with IBD care and improve long-term outcomes.

### Limitations

The first limitation is that our dataset is small and the independent test set comes from the same center, which means that in the future, we need to conduct extensive external validation with data from other centers. The second issue is that the endoscopic activity classes are unevenly distributed, which may lead to calculation biases. However, we attempted to minimize these biases by using the SMOTE function in R.

## 5. Conclusions

To sum up, employing ML models that incorporate clinical and laboratory variables can be a valuable non-invasive method for estimating EDA in patients with long-standing UC. The development and validation of enhanced ML techniques could assist in identifying personalized treatment plans and follow-up strategies and could result in more frequent monitoring of subclinical UC disease activity with fewer invasive procedures, reduced exposure to potential risks and increased patient comfort.

## Figures and Tables

**Table 1:** Parameters used to train the ML models with the caret::train function.

method	“rf”/“mlpML”
preProcess	c(“scale”, “center”)
trControl	trainControl(method = “repeatedcv”, number = 10, repeats = 10, sampling = “smote”)

**Table 2:** Selected parameters for all patients and each endoscopic activity group.

	All	Endoscopic Remission/Mild Disease	Endoscopic Relapse		
			Total	Moderate Disease	Severe Disease
Number of records	187	38	149	72	77
Gender (male:female)	110 (59%):77 (41%)	16 (42%):22 (58%)	94 (63%):55 (37%)	37 (51%):35 (49%)	57 (74%):20 (26%)
Age (years)	45 (23.5)	52.5 (23.5)	44 (23)	44 (25.25)	44 (23)
WBC count/µL	7800 (3585)	7600 (2657.5)	7960 (3560)	7580 (3512.5)	8390 (4780)
Platelet count/µL	291,000 (121,500)	237,500 (102,250)	297,000 (137,000)	286,000 (102,500)	303,000 (153,000)
Fibrinogen mg/dL	390.1 (88.5)	360 (63)	394 (99)	390.5 (68.5)	400 (124)
CRP mg/dL	1 (2.6)	0.52 (1.08)	1.1 (3.69)	1.05 (2.56)	1.09 (4.78)
Oral corticosteroids (yes/no)	55:132	4:34	51:98	16:56	35:42
IBDQ score	137 (53)	161 (50.25)	133 (52)	140 (37.5)	120 (54)

**Table 3:** The results of applying Kruskal–Wallis method to determine the continuous variables that were significantly related to the endoscopic activity.

Parameter	Chi Square	df	*p*
IBDQ	28.793	3	<0.001
Smoking pack years	6.679	3	0.083
Relapses per year	5.709	3	0.127
Hemoglobin	1.173	3	0.760
Hematocrit	0.805	3	0.848
MCV	1.023	3	0.796
MCHC	0.169	3	0.983
Platelet count	17.316	3	<0.001
WBC count	10.905	3	0.012
ESR	3.733	3	0.292
Fibrinogen	12.45	3	0.006
CRP	9.375	3	0.025
Total proteins	7.387	3	0.061
Albumin	3.559	3	0.313

**Table 4:** The outcomes of applying Pearson’s chi-squared test to determine the categorical variables that were significantly correlated to the endoscopic activity.

Medication	Chi Square	df	*p*
Topical 5-ASA	8.244	9	0.51
Oral 5-ASA	18.717	15	0.227
Intravenous corticosteroids	12.323	9	0.196
Oral corticosteroids	19.635	9	0.02
Azathioprine	2.254	6	0.895
Infliximab	1.603	3	0.659
Adalimumab	3.229	3	0.358
Antibiotics	7.146	6	0.308

**Table 5:** The performance metrics of the first RF and MLP classifiers (IBDQ excluded as a predictor).

	Random Forest				Multilayer Perceptron			
	Train Set		Test Set		Train Set		Test Set	
	Actual Values		Actual Values		Actual Values		Actual Values	
Predicted values	Remission	Activity	Remission	Activity	Remission	Activity	Remission	Activity
Remission	31	8	5	4	20	27	6	6
Activity	0	111	2	26	11	92	1	24
Accuracy	95%		84%		75%		81%	
95% CI	(0.8976, 0.9767)		(0.6799, 0.9381)		(0.6693, 0.8141)		(0.6484, 0.9204)	
Sensitivity	100%		71%		65%		86%	
Specificity	93%		87%		77%		80%	
PPV	SE80%		56%		43%		50%	
NPV	100%		93%		89%		96%	
AUC	0.9905		0.8357		0.7934		0.8286	

**Table 6:** The performance metrics of the second RF and MLP classifiers (IBDQ included as a predictor).

	Random Forest				Multilayer Perceptron			
	Train Set		Test Set		Train Set		Test Set	
	Actual Values		Actual Values		Actual Values		Actual Values	
Predicted values	Remission	Activity	Remission	Activity	Remission	Activity	Remission	Activity
Remission	31	7	6	3	27	22	7	7
Activity	0	112	1	27	4	97	0	23
Accuracy	95%		89%		83%		81%	
95% CI	(0.9062, 0.981)		(0.7458, 0.9697)		(0.7564, 0.8835)		(0.6484, 0.9204)	
Sensitivity	100%		86%		87%		100%	
Specificity	94%		90%		82%		77%	
PPV	82%		67%		55%		50%	
NPV	100%		96%		96%		100%	
AUC	0.9999		0.9095		0.8978		0.8714	

**Table 7:** The performance metrics of the third RF and MLP classifiers to predict endoscopic severity (IBDQ excluded as a predictor).

	Random Forest						Multilayer Perceptron					
	Train Set			Test Set			Train Set			Test Set		
	Actual Values			Actual Values			Actual Values			Actual Values		
Predicted values	Mild	Moderate	Severe	Mild	Moderate	Severe	Mild	Moderate	Severe	Mild	Moderate	Severe
Mild	49	0	0	3	2	1	34	15	16	7	5	3
Moderate	0	50	0	4	5	4	11	21	7	2	5	5
Severe	0	0	50	2	5	11	4	14	27	0	2	8
Accuracy	100%			51%			55%			54%		
95% CI	(0.9755, 1)			(0.344, 0.6808)			(0.4668, 0.6318)			(0.3692, 0.7051)		
Multiclass AUC	1			0.6425			0.695			0.6883		

**Table 8:** The performance metrics of the fourth RF and MLP classifiers to predict endoscopic severity (IBDQ included as a predictor).

	Random Forest						Multilayer Perceptron					
	Train Set			Test Set			Train Set			Test Set		
	Actual Values			Actual Values			Actual Values			Actual Values		
Predicted values	Mild	Moderate	Severe	Mild	Moderate	Severe	Mild	Moderate	Severe	Mild	Moderate	Severe
Mild	49	0	0	8	4	1	40	23	11	7	7	1
Moderate	0	50	0	0	5	3	5	6	4	1	2	1
Severe	0	0	50	1	3	12	4	21	35	1	3	14
Accuracy	100%			68%			54%			62%		
95% CI	(0.9755, 1)			(0.5021, 0.8199)			(0.4601, 0.6254)			(0.4476, 0.7754)		
Multiclass AUC	1			0.7614			0.6976			0.7284		

## Data Availability

The data presented in this study are available on request from the corresponding author.
